# Current approaches in the surgical treatment of liver hydatid disease: single center experience

**DOI:** 10.1186/s12893-019-0553-1

**Published:** 2019-07-17

**Authors:** Mehmet Bayrak, Yasemin Altıntas

**Affiliations:** 1 Department of General Surgery , Ozel Ortadogu Hospital, Ziyapasa Mahallesi 67055 Sokak no:1, Adana, Turkey; 2 Department of Radiology , Ozel Ortadogu Hospital, 01360 Adana, Turkey

**Keywords:** Hydatid cyst, Liver, Laparoscopy, ERC, Biliary communication

## Abstract

**Background:**

Liver hydatid disease is a common benign condition in many countries. Compared to open surgery, laparoscopic treatment can play an important role in improving the post-operative recovery, reducing the morbidity and recurrence rate of these patients.The purpose of this study is to show that the laparoscopic method is effective and safe in the treatment of liver hydatid cysts compared to open surgery, even in large cysts.

**Methods:**

All consecutive cases surgically managed for liver hydatid cyst from 7 January 2008 and 15 January 2010 in our institution were included in this study.The surgical approach (laparoscopic or open) and operative strategy, as well as operative and prognostic outcomes, were analyzed. Cyst size, type, location, presence of biliary tract communication, radiological findings, duration of hospitalization, recurrence and postoperative morbidity were analysed and compared retrospectively.

**Results:**

A total of 60 patients were included in the study.A total of 23 patients underwent open surgery, and 37 patients underwent laparoscopic surgery.Operation types of laparoscopic surgery were as follows: partial pericystectomy (12patients), total cystectomy(2 patients), partial pericystectomy+total cystectomy(7patients) and cystectomy(16patients).The surgical procedures chosen for open treatment of the residual cavity were partial pericystectomy and omentoplasty(17cases), total pericystectomy(3cases) and partial and total pericystectomy(3cases).Cysto-biliary communication was found in 9 patients. A total of 10 patients underwent preoperative endoscopic retrograde cholangiography, and one patient underwent postoperative endoscopic retrograde cholangiography.There was a progression of hypernatremia in 1 patient, wound infections in 3 patients, and perioperative hemorrhage in 3 patients. There were no statistically significant differences concerning age(*p* = 0.344), gender(*p* = 0.318), ASA classification(*p* = 0.963), Gharbi classification(*p* = 0.649) whereas there were significant differences related to cyst location(*p* = 0.040) and size(*p* = 0.022) in patients undergoing laparoscopic and open surgery.

Postoperative temporary biliary fistulas were observed in 2 patients undergoing open surgery. Patients undergoing laparoscopic surgery had the advantages of shorter hospital stays and operation times, less blood loss, faster recovery, and lower wound infection rates. Recurrences were detected in 2.7% of patients undergoing laparoscopic surgery and 4.7% of those undergoing open procedures.

**Conclusion:**

Compared to open surgery in the treatment of liver hydatid cysts, we have shown that laparoscopic method can be safely performed even in large cysts and/or cysto-biliary communication.

## Background

Hydatid disease is endemic in the Mediterranean, South America, Far East, Central Asia and Eastern Europe. However, it is also frequently observed in nonendemic countries because of the increase in global travel [1]. Approximately 4,000 diagnoses of hydatid disease have been recorded annually in Turkey [2]. Symptoms typically develop as a consequence of the compression of adjacent structures or viscera that result from surrounding inflammation, or from the rupture of the cyst into the bile duct, pleural space or peritoneal cavity. The liver is most commonly affected, with the involvement of the right lobe in 55–80% of patients. The clinical presentation of intrabiliary rupture (IBR) can range from asymptomatic to jaundice, cholecystitis, cholangitis, liver abscess, pancreatitis, and septicemia, depending on the size of the cysto-biliary communication. In clinical practice, it is generally agreed that endoscopic retrograde cholangiography (ERC) is indicated for patients with biliary fistulae and jaundice, as well as for preoperative IBR that is suspected clinically, biochemically or radiologically [3, 4].

According to The American College of Gastroenterology Guidelines; surgery, either laparoscopic or open, based on available expertise, is recommended in complicated hydatid cysts with multiple vesicles, daughter cysts, fistulas, rupture, hemorrhage, or secondary infection [5].

Laparoscopic liver hydatid resections have steadily increased among surgeons from both endemic and nonendemic areas [6]. Although early reported laparoscopic treatments of liver hydatid disease were confined to simple drainage, more advanced laparoscopic methods are now possible, including pericystectomy and even segmentectomy in selected cases [6–8].

We present the surgical treatment of hydatid disease patients at our clinic. This to show that the laparoscopic method is reliable even in large cysts when compared with open surgery in the treatment of liver cyst hydatid.

## Methods

All patients treated either by laparoscopic or open surgery for liver hydatidosis at our institution between January 2008 and January 2018 were included in the study. Each patient’s medical record was reviewed retrospectively for results of the physical examination, serum biochemistry, abdominal ultrasound (US), abdominal computed tomography (CT), magnetic resonance imaging (MRI) or magnetic resonance cholangiopancreatography (MRCP). The Gharbi classification system was used to stage the hydatid disease [9]. The liver segments were grouped as near to the hilum (segments I, III, IVb, V, and VI) and remotely distant (segment II, IVa, VII, and VIII) with modification of classification by Dziri et al. We designated the segments nearest to the hilum as central and those farther away as peripheral [8, 10]. Written approval was obtained from Cukurova University Faculty of Medicine Clinical Ethical Board.

All patients were treated with albendazole (10 mg/kg) 7 days before surgery, and this medication was continued postoperatively for two months. MRCP and/or ERC were used preoperatively for patients with jaundice, cholangitis, dilated biliary ductal system, hydatid elements evidence in the bile ducts or elevation of serum liver transaminases.

### Operative strategy

**Fig. 1:**
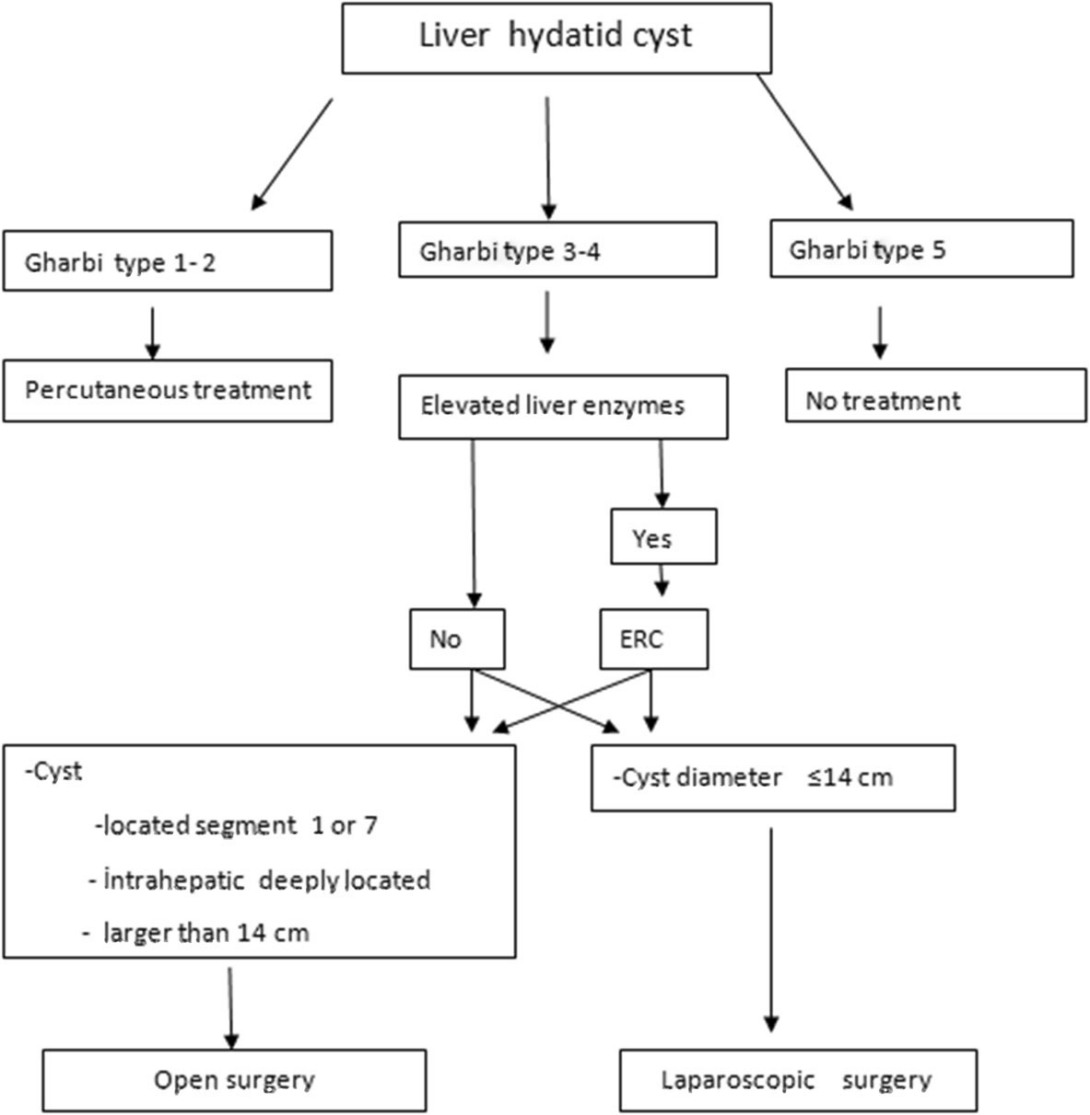
Treatment algorithms of liver hydatid cysts

The surgical strategy was chosen according to the number, size, location and complicated presentation of the cyst(Fig. 1). We follow the scientific guidelines for operative strategy.

**Fig. 2:**
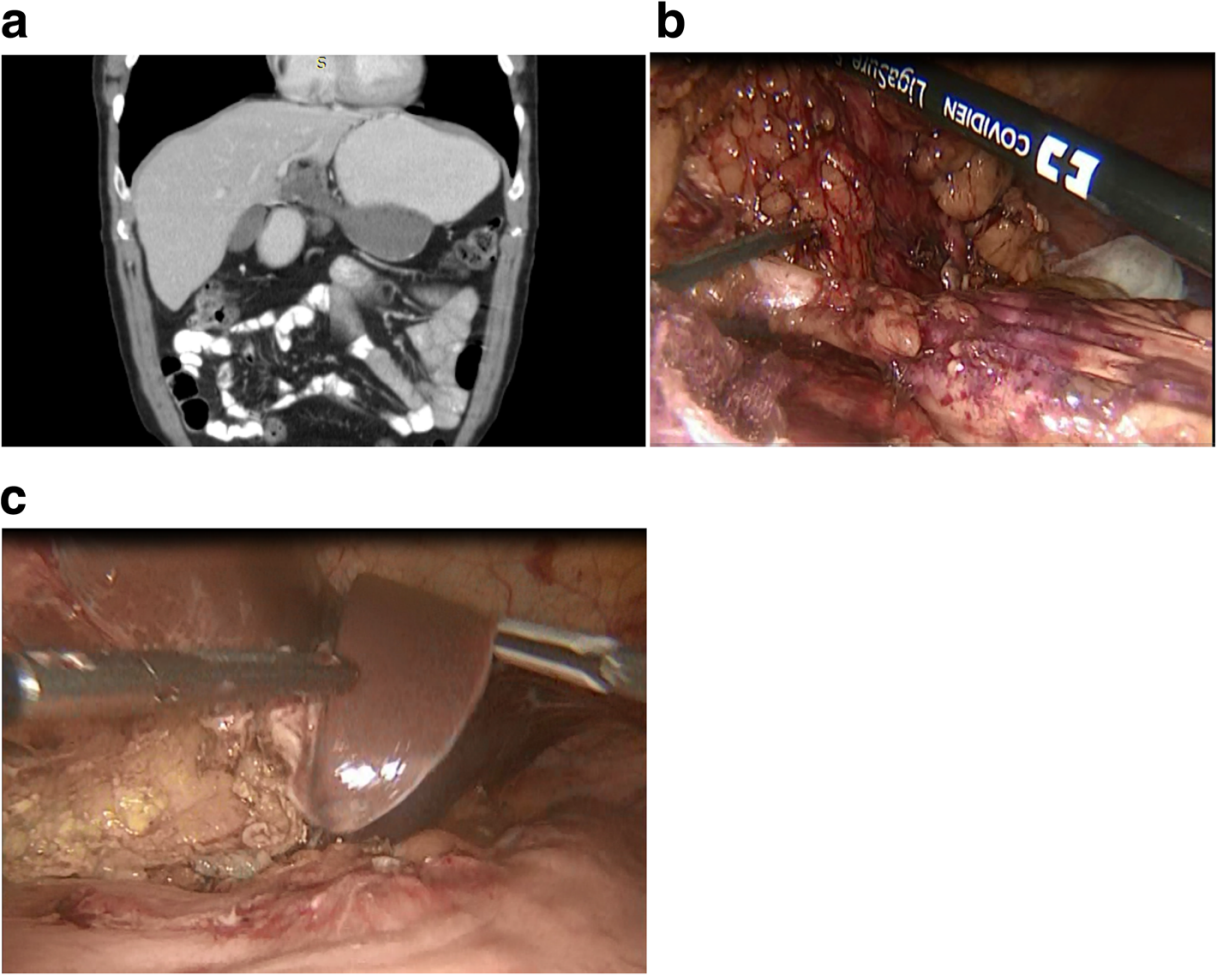
**a** CT imaging of type 3 cystic hydatid cyst with exophytic distension from the left lobe of the liver, compressing the stomach posteroinferiorly and operative figures of partial pericystectomy in this case (**b**); the appearance at the end of the operation (**c**)

**Fig. 3:**
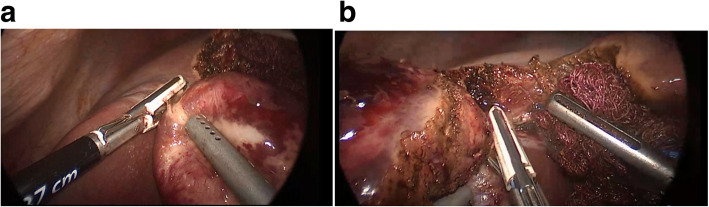
Operation figures of the case which total cystectomy was performed (**a**, **b**)

The open or laparoscopic conservative resections of hydatid cysts included cystectomy and partial pericystectomy. Cystectomy involved aspiration of the cyst, installation of a scolicidal substance to kill the parasite, evacuation of the parasitic material and then preservation or partial resection of the pericyst (partial pericystectomy) (Fig. 2 b, c). Concerning treatment of the residual cavity, omentoplasty was done in partial pericystectomy. Radical surgical procedures included total cystectomy (Fig. 3a, b) (removal of the cyst and 1–2 cm of normal liver parenchyma around the cyst) or total cystectomy (complete removal of the cyst).

Only the following aspects were considered as exclusion criteria for laparoscopic surgery: liver hydatid cysts located in segment 1 or 7 of the liver (Couinaud’s segmentation), deeply located intraparenchymal cysts or cysts larger than 14 cm in size.

Patients manageable by percutaneous treatment were excluded by the study.

### Surgical techniques

#### Techniques for laparoscopic hydatid cyst surgery

In all operations, prophylactic antibiotics (cefazolin 1 g) were given to patients 20 min before the general anesthesia was performed. A 10-mm trocar was inserted into the supraumbilical region, and a 30° telescope was placed. Then, a 10-mm trocar and two 5-mm trocars were placed at the epigastrium according to cyst localization. Carbon dioxide pneumoperitoneum was created, and the intraabdominal pressure was adjusted to 12 mmHg. After routine exploration, gauze pads impregnated with hypertonic saline (20% sodium chloride) were placed on the cyst protruding from the liver, Morrison’s pouch, and subhepatic space. The cyst contents were evacuated using a Veress needle, and the cyst was refilled with hypertonic saline. The hypertonic solution remained for 5 min followed by aspiration. The cyst wall was punctured with a perforator grinder aspirator, and daughter vesicles in the cyst were aspirated completely. The protruded pericyst wall was excised using LigaSure™ (Valleylab, Boulder, CO, USA). The excised pericyst wall, germinal membrane, and gasses were removed through the 10-mm trocar using an endobag. Subsequently, cyst residues, biliary rupture and or hemorrhage were examined in the cystic cavity by laparoscopy. When a communication between the cyst wall and the biliary tract was observed, it was stitched with non-absorbable sutures, and a drain (20-F Nelaton drain) was placed according to the location of the cyst. A sufficient portion of greater omentum was excised and moved and placed in the cyst cavity. The part of omental fat was secured to the excised side of cyst wall using a helical fastener (Pro Tack, Auto Suture, Norwalk, Connecticut, USA).

#### Techniques for open hydatid cyst surgery

We usually perform laparotomy with right subcostal or midline incision based on the location of the cyst.

A 20% hypertonic saline solution was used to deactivate the cyst content. To prevent secondary peritoneal hydatidosis, the puncture site was covered with hypertonic saline solution-soaked gauzes before any maneuver on the hydatid cyst was performed. After 5 min, the cyst content was aspirated. Starting from the puncture site, cystotomy was performed, with the extraction of the germinal membrane and daughter vesicles. Then a sufficient part of omental fat was sutured to the cyst wall.

### Statistical method

The SPSS 23.0 package program was used for statistical analysis of the data. Categorical measurements are presented as numbers and percentages, and continuous measurements are presented as means and standard deviations (median and minimum-maximum where necessary). The chi-square or Fisher’s exact tests were used to compare categorical variables. The Mann-Whitney U test was used to compare continuous variables with variables such as surgery type. The statistical significance level was considered to be less than 0.05 in all tests.

## Results

### Results of the Total cohort

A total of 60 patients who had been operated for hydatid cyst disease in our clinic between January 2008 and January 2018 were included in the study. Twenty-three patients were treated by open surgery, and 37 patients were treated laparoscopically. The average age of our patients was 43.6 (range 14–88) years. There was a single cyst in 37 patients (61.7%) and several cysts in 23 patients (38.3%). Preoperative demographic findings and diagnostic methods are shown in Table 1. The cyst localization, size, localization lobe and Gharbi classification are shown in Table 2. The average cyst diameter was 13.6 ± 2.8 cm.

Because of deranged levels of ALT and AST a total of 10 patients (16.7%) underwent preoperative ERC. Three of these patients had bilirubin levels above normal, and three patients had cholangitis. Biliary tract dilatation was detected radiologically in these patients and a daughter vesicle in the intrahepatic biliary tract was detected in one patient, and a daughter vesicle in the choledochus was detected in another(Table 1).

**Table 1:** Patients demographic and diagnostic findings

	n	%
Gender		
Male	20	33.3
Female	40	66.7
Age*	43(14–88)	
US	60	100
CT	60	100
MR	8	13.3
MRCP	6	10
Elevated Liver enzymes	10	16.7
Preoperative ERC	10	16.7
Postoperative ERC	1	1.7
Temporary biliary fistula	2	3.3
Cysto-biliary communication	10	16.7

*Median(Min-Max)

**Table 2:** Cyst characteristics

	n	%
Cyst location		
Central	38	63.3
Peripheral	15	25.0
Central and peripheral	7	11.7
Cyst lobe		
Right	52	86.7
Left	3	5.0
Right and left	5	8.3
Cyst type#		
Type 1	2	3.3
Type 2	48	80.0
Type 3	10	16.7
Cyst number		
Multiple	23	38.3
Single	37	61.7
Average* cyst size	14(8–20)	

*Median(Min-Max**)**, #: Gharbi classification

**Table 3:** The variables whose statistically significant relationship with cyst size was detected

	ALT-AST-ALP		
	Normal	High	
	Median (min-max)	Median (min-max)	P
Cyst size	13 (8–20)	16 (14–20)	0.0001
	Biliary tract dilatation		
	Yes	No	
Cyst size	16 (14–20)	13 (8–20)	0.001
	Temporary biliary fistula		
	Yes	No	
Cyst size	19 (18–20)	13 (8–20)	0.005
	Cysto-biliary communication		
	Yes	No	
Cyst size	15 (14–20)	13 (8–20)	0.005

p: Mann-Whitney U test

**Table 4:** Comparison of laparoscopic and open surgery

	Laparoscopic surgery Median (min-max)	Open surgery Median (min-max)	p
Number of patients	*n* = 37	*n* = 23	
Age (years)	38 (15–75)	46 (14–88)	0.344
Gender			
Males	11(29.7)	9(39.1)	0.318
Females	26(70.3)	14(60.9)	
ASA classification			
1	17(45.9)	10(43.4)	
2	13(35.1)	8(34.7)	0.963
3	7(19.0)	5(21.9)	
Gharbi classification			
Type 2	1(2.7)	1(4.3)	
Type 3	31(83.8)	17(73.9)	0.649
Type 4	5(13.5)	5(21.8)	
Cyst location			
Central	19(51.4)	19(82.6)	**0.040**
Peripheral	13(35.1)	2(8.7)	
Central & peripheral	5(13.5)	2(8.7)	
Cyst size	13 (8–20)	15 (10–20)	**0.022**
Cysto-biliary communication	7(18.9)	2(8.7)	0.460
Operation methods			
Partial pericystectomy	12 (32.4%)	17 (73.9%)	
Total cystectomy	2 (5.4%)	3 (13.0%)	0.001
Cystectomy	16 (43.2%)	0 (0%)	
Partial pericystectomy +total cystectomy	7 (18.9%)	3 (13.0%)

**Table 5:** Outcomes of operation methods

	Partial pericystectomy	Total cystectomy	Cystectomy	Partial pericystectomy +total cystectomy	p
Operation time(min)	5(3–15)	5(3–7)	3(2–10)	4(3–15)	0.016
Blood loss(ml)	69(46–83)	73(60–220)	**61(40–70)**	**59(49–180)**	0.007
Length of hospital stay(days)	18(9–45)	20(8–48)	12(9–35)	14(10–46)	0.110
Morbidity					
Surgical site infection	4(13.8)	0(0.0)	0(0.0)	0(0.0)	0.205
Temporary biliary fistula	2(6.9)	0(0.0)	0(0.0)	0(0.0	0.530
Periop. bleeding	1(3.4)	1(20.0)	0(0.0)	1(10.0)	0.274
Recurrence	1(3.4)	0(0.0)	1(6.3)	0(0.0)	0.816

**Table 6:** Comparison of operative results and outcomes of laparoscopic and open surgery

	Laparoscopic surgery Median (min-max)	Open surgery Median (min-max)	p
Operation time(min)	50(35–70)	74(39–95)	**0.0001**
Blood loss(ml)	60(40–220)	75(65–83)	**0.0001**
Length of hospital stay(days)	4(2–15)	7(3–15)	**0.0001**
ERC			
Preoperative	7(18.9)	3(13.0)	***0.012***
Postoperative	0(0.0)	1(4.3)	
Morbidity			
Surgical site infection	0(0.0)	3(13.0)	0.052
Temporary biliary fistula	0(0.0)	2(8.7)	0.143
Periop. Bleeding	2(5.4)	1(4.3)	1.000
Recurrence	1(2.7)	1(4.3)	1.000
Mortality	0	0	1.000

A biliary fistula was observed postoperatively in 2 patients. Fistula output was approximately 60–80 ml per day. The fistula closed spontaneously on the 4th day in one of these patients. The other patient underwent postoperative ERC on the 7th day and the fistula closed on the 9th day. One patient had an abscess associated to the cyst and underwent emergency surgical treatment. Three patients developed wound infections while 3 patients experienced perioperative hemorrhage. The hemorrhage was controlled perioperatively without the need for blood transfusion. In a patient underwent open surgery with a giant cyst, hypernatremia developed due to the excessive use of 20% hypertonic saline solution. This was treated with rehydration. Surgical site infection and temporary biliary fistula were not observed in laparoscopic surgery, but in 3 and 2 patients respectively in open surgery. Perioperative bleeding was observed in 2 patients in laparoscopic surgery and 1 patient in open surgery. There was a postoperative recurrence in one patient in both laparoscopic and open surgery, detected in 18 and 24 months, respectively. The average follow-up duration was 21.3 ± 13.1 months.

We categorized the size of the cysts in two groups as 1–9 cm and 10 cm or larger. We determined that transaminase levels, biliary tract dilatation, and cysto-biliary communication rates significantly increased as the cyst size increased. The cyst size was larger in patients with postoperative biliary fistula (Table 3).

The operation methods and types are presented in Table 4.

**Fig. 4:**
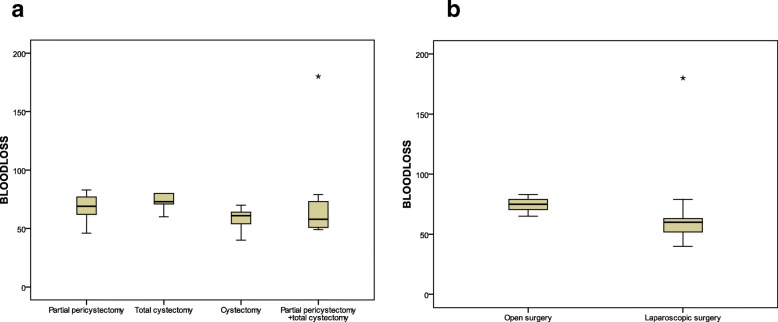
**a** Blood loss outcomes of the operation methods, **b** comparison of blood loss outcomes between laparoscopic and open surgery

The outcomes of the operation methods (partial pericystectomy or total cystectomy or combined) are shown in Table 5 (Fig. 4a).

### Comparison between open and laparoscopic approach

We performed open surgery on 23 patients and laparoscopic surgery on 37 patients. Since 2012, we have changed our operation type selection to laparoscopic surgery.

Operation types of laparoscopic surgery were as follows: omentoplasty was performed in all partial pericystectomies (12 patients). Two patients underwent total cystectomy, and seven patients underwent laparoscopic partial pericystectomy + total cystectomy. In 16 patients undergoing cystectomy, omentoplasty was not performed; and a drain was placed.

The surgical procedures chosen for open treatment of the residual cavity were partial pericystectomy and omentoplasty (17 cases), total pericystectomy (3 cases) and partial and total pericystectomy (3 cases). A drain was then placed into the subhepatic or subdiaphragmatic space. All patients underwent omentoplasty except total cystectomy in open surgery.

There were no statistically significant differences concerning age, gender, ASA classification, anatomic site or Gharbi classification in patients undergoing laparoscopic and open surgery (Table 4).

The mean cyst sizes in patients who underwent open and laparoscopic surgery were 14.6 ± 2.5 cm and 12.9 ± 2.8 cm, respectively. When the patients were compared concerning cyst size, cysts were significantly smaller in laparoscopic surgery patients (*p* = 0.022). There was no significant difference between the two types of operation regarding cysto-biliary communication. There was a considerable difference between the two kinds of operation concerning cyst localization. Laparoscopic surgery was preferred for patients with more peripheral involvement, and open surgery was preferred for patients with more centrally located cysts. Because we preferred open surgery in large cysts (over 14 cm), average cyst size was greater in patients who underwent open surgery than in laparoscopic surgery.

The mean duration of operation was 72.4 ± 12.8 min for open surgery patients and 49.8 ± 10.4 min for laparoscopic surgery patients (*p* = 0.0001). Blood loss was 74.4 ± 5.2 ml for the open surgery patients and 60.8 ± 22.3 ml for the laparoscopic surgery patients (*p* = 0.005) (Fig. 4b). The duration of hospitalization was 4.3 ± 2.5 days and 6.8 ± 2.7 days in patients undergoing laparoscopic surgery and open surgery, respectively *p* = 0.01 (Table 6).

No wound infection was observed in any patient who underwent laparoscopic surgery while wound infection occurred in 3 open surgical patients.

All advantages of minimally invasive surgery such as shorter hospital stay, very low wound infection, shorter operation time and less blood loss were observed in laparoscopic surgery of hydatid cysts(Table 5).

No biliary fistula was observed in any patient who underwent laparoscopic surgery; while, it was detected in 2 patients undergoing open surgery.

Recurrences were detected in 2.7% (1 patient) of patients undergoing laparoscopic surgery and 4.7% (1 patient) of those undergoing open surgical procedures (*p* = 1,000). No deaths were observed with either operation type.

## Discussion

The symptoms of hydatid disease vary according to cyst localization, size, type and affected organ. Although most liver hydatid cysts are asymptomatic, the most common symptom is right upper quadrant pain and hepatomegaly. In most patients with liver hydatid cysts, there are no complications at diagnosis and no problems with treatment. However, for the diagnosis and treatment of complicated cases, the roles of surgery, interventional radiology, and therapeutic endoscopists is essential.

The most common complications of liver hydatid cyst are rupture into intrahepatic biliary tracts, spread to other organs and pressure and infection of the biliary system. Intrabiliary rupture ranges from 2 to 42% in various series [11–13].

Ultrasonography is the preferred radiological method for cyst diagnosis because of its low cost and high specificity and sensitivity. We recently encountered more complicated cases due to the increase in percutaneous treatment options. CT, MRI, and MRCP have gained importance because of their ability to reveal characteristics of the biliary/vascular system and cyst as well as the relations with adjacent structures.

The pressure in the cyst can be 80 cmH2O and is usually 35 cmH2O pressure. This is an indicator of cyst viability. Cysts may cause compression or obstruction of the biliary system. In addition, they may biochemically and radiologically cause [14, 15].

Rupture into the biliary system is the most frequent complication of liver hydatid disease. The communication between the cyst wall and the ductal system is classified as major or minor. Minor communication is often silent, while major complications produce clinical and radiological symptoms. Larger cysts have been demonstrated to be more susceptible to cysto-biliary communication [16]. In the present study, we also found that there was a significant relationship between cyst size with cysto-biliary communication and with biliary tract dilatation. Kayaalp et al. reported that there were more cysto-biliary communications in centrally located cysts than with peripheral cysts [15]. However, we observed no significant differences regarding cysto-biliary communication with cyst localization (central or peripheral), cyst number (single, multiple) or cyst type. Intrabiliary rupture may cause clinical symptoms, including cholecystitis, cholangitis, liver abscess, pancreatitis, and sepsis depending on the size of cysto-biliary communication. In our study, there was cholangitis in 3 patients, cholecystitis in 2 patients and radiologically apparent biliary tract dilatation in 10 patients. Patients underwent preoperative diagnostic/therapeutic ERC. There was biliary tract dilatation due to cystic pressure in 7 patients, daughter vesicle in the intrahepatic biliary tract in one patient and a daughter vesicle in the choledochus in one patient. When in doubt regarding cysto-biliary communication, ERC should be performed before the operation. We performed preoperative ERC in patients with elevated liver enzymes, cholangitis, radiologically biliary duct dilatation and/or biliary leakage. It was reported in many series that preoperative ERC was successful in more than 80% of endoscopic sphincterotomies for the removal of biliary daughter vesicles, as well as for placement of nasobiliary drainage and/or stents for the treatment of complicated liver hydatid cysts. Therefore, treatment of these complicated cases has been made possible with the laparoscopic method [3, 4, 17–19]. Since the removal of hydatid cysts in the choledochus and intrahepatic biliary tract with ERC is ensured, laparoscopic intervention can be performed instead of open surgery and removal of cyst by choledochotomy. In our study, daughter vesicles were also successfully removed from the intrahepatic and choledochus in two separate patients, and these two patients underwent laparoscopic surgery.

There is a broad spectrum of treatment options, including systemic chemotherapy, percutaneous treatment with/without medical treatment and conventional or laparoscopic surgery. Chemotherapy has been suitable for diffuse diseases or patients with contraindications to operation. Albendazole has been preferred as a chemotherapeutic agent, and its typical dosage is 10–15 mg/kg/day [20]. Radical or conservative surgery is the cornerstone of the treatment of liver hydatid cyst. However, for noncomplicated type, I and type II cysts, for which surgery is contraindicated, medical treatment with percutaneous drainage is an excellent alternative to surgery. In some series, percutaneous procedure was also performed in patients with type III cysts [21–25].

Surgical methods remain the first choice for type III and type IV cysts opening into the biliary system and peritoneal cavity [23, 25]. During the last five years of our 10-year series, we switched to laparoscopic surgery from open surgery for the surgical treatment of liver hydatid cysts. Laparoscopic surgery was not immediately accepted initially due to concerns that intraperitoneal dissemination, hemorrhage and recurrence rates may be higher than for conventional surgery. In our study, however, no intraperitoneal spillage was seen in any of the patients undergoing laparoscopic surgery. Perioperative blood loss was significantly lower in patients undergoing laparoscopic surgery. Recurrence rates were also lower in the laparoscopy group. None of the patients who underwent laparoscopic surgery were re-operated by open surgery. As our experience increased, we performed more difficult cases using the laparoscopic approach (Fig. 3 a, b, c).

Tuxun et al. reported morbidity, mortality and recurrence rates of 15.07, 0.22, and 1.09%, respectively in a review analysis of 914 cases, suggesting that laparoscopy was reliable in selected patients [6].

We performed hydatid cyst treatment using open surgery in 23 patients and laparoscopic surgery in 37 patients. If the cyst was peripherally localized, total cystectomy or hepatic resection was preferred because the recurrence rate was lower. In our study, we performed total cystectomy using open surgery in 3 patients and using laparoscopy in 2 patients, as well as multiple and/or partial pericystectomy+total cystectomy due to various lobe localizations using open surgery in 3 patients and using laparoscopy in 7 patients. There were no recurrences in any of the patients undergoing total cystectomy. There was no intraperitoneal spread in any patient. The most frequently performed operation type for cysts with intraparenchymal localization was partial pericystectomy and omentoplasty in open surgery, and cystectomy and partial pericystectomy in laparoscopic surgery. We performed laparoscopic cystectomy as the operation type for cysts that were mildly or moderately located in the liver parenchyma. We preferred the partial pericystectomy operation type if the cyst was close to the liver capsule or protruded from the liver parenchyma in both open and laparoscopic surgery. In this study, according to our operative strategy, we found no significant differences regarding operative or prognostic outcomes between these operation methods.

Omentoplasty absorbs residual fluid in the cyst pouch and stimulates macrophage migration to the surgical region [10]. All patients who underwent partial pericystectomy in laparoscopic and open surgery underwent omentoplasty.

One of the most critical advantages of laparoscopic surgery is that the laparoscope can enter into the cyst cavity and allow detailed inspection. Because it has a threefold larger image, it shows better bile duct leakage within the cyst. If biliary leakage is present, it can be treated with clips or sutures.

Our study showed that patients undergoing laparoscopic surgery had the advantages of shorter hospital stays, shorter operation times, less blood loss, a better cosmetic effect, faster recovery, and lower wound infection rates.

A drain was usually placed to prevent biloma, abscess and biliary peritonitis. If biliary drainage lasted ten days or more, it was called a biliary fistula. If biliary drainage had a low output (< 100 ml/day) or the drainage amount decreased, it could be expected to close spontaneously. If it does not decrease and/or it increases, postoperative ERC should be performed [15, 26, 27]. There was biliary drainage (< 100 ml/day) in two patients in our series. The drainage was stopped in one patient on the 4th day. Postoperative ERC was performed in the other patient since the drainage amount did not decrease, and the drainage was stopped.

Although there was no intrahepatic subcapsular hematoma in any of our patients; the development of subcapsular hematoma in patients suffering from abdominal pain after surgical operation of the liver hydatid cyst should be kept in mind especially if anticoagulant drug and non-steroidal anti-inflammatory drug use or capsule laceration present in the operation.

Recurrence is an important problem in hydatid cyst surgery. The recurrence rate of open surgery has been reported as 0–4% in various studies. However, the cumulative recurrence rate of laparoscopic surgery has been reported to be 1.1% [6]. In our study, the recurrence rate was 4.3% in patients with open surgery, and there was one (2.7%) recurrence in patients with laparoscopic surgery.

At postoperative follow-up, indirect hemagglutination (IHA) test and a Ge radioallergosorbent test (RAST) should be used together with USG or other radiological modalities [28].

The limitation of this study was its retrospective nature.

## Conclusion

Given the well-known advantages of minimally invasive surgery, the laparoscopic approach replaced the conventional surgery for the treatment of liver cyst hydatidosis.

In our study cysts size and location were significanlty related to the surgical technique. So we would conclude that laparoscopic surgery is safe and feasible in the majority of cases, but in case of large size and intra parenchymal cyst, open surgery is more commonly performed. As our experience increased larger cysts or deeper located intraparenchymal cysts, the presence of biliary communication can be treated with the laparoscopic technique in accordance with the open method of surgical intervention. We suggest laparoscopic cystectomy or partial periscystectomy as the operation method for the intraparenchymal cyst.

Since there is no difference in outcome between partial pericystectomy and total cystectomy in our cohort, and because it is already a benign condition, radical laparoscopic surgery should be reserved only in peripheral cysts.

## Data Availability

The datasets used and/or analyzed during the current study are attached as supplementary files.

## Abbreviations

ALT: Alanine aminotransferase
ASA: *American Society of Anesthesiologists*
AST: Aspartate aminotransferase
CT: Computed tomography
ERC: Endoscopic retrograde cholangiography
IBR: Intrabiliary rupture
IHA: Indirect hemagglutination
MRCP: Magnetic resonance cholangiopancreatography
MRI: Magnetic resonance imaging
RAST: Radioallergosorbent test
US: Ultrasound
